# IgE in the Pathogenesis of SLE: From Pathogenic Role to Therapeutic Target

**DOI:** 10.3390/antib9040069

**Published:** 2020-12-08

**Authors:** Yasmine Lamri, Nicolas Charles

**Affiliations:** 1 Centre de Recherche sur l’Inflammation, CNRS ERL8252 Faculté de Médecine Site Bichat, Université de Paris, INSERM UMR1149, 16 Rue Henri Huchard, F-75018 Paris, France; yasmine.lamri@inserm.fr; 2 Laboratoire d’Excellence INFLAMEX, Université de Paris, F-75018 Paris, France

**Keywords:** autoantibodies, isotypes, lupus, SLE, Fc receptors, IgE, basophils, plasmacytoid dendritic cells

## Abstract

Systemic lupus erythematosus (SLE) is a multifactorial chronic autoimmune disease, marked by the presence of autoantibodies to nuclear antigens belonging to different isotype classes. For several years, IgE antibodies have been incriminated in the development of allergic diseases and parasitic infections and different anti-IgE therapies have been developed to encounter the pathogenic role of IgE in these pathologies. Recently, multiple studies showed the presence of elevated total IgE levels and demonstrated a pathogenic role of autoreactive IgE in SLE. This review aims to summarize the findings incriminating IgE and autoreactive IgE in the pathophysiology of SLE, to describe their functional outcomes on their targeted cells as well as to discuss different IgE-related therapeutic modalities that emerged and that may be beneficial for SLE patient care.

## 1. Introduction

Systemic lupus erythematosus (SLE) is a chronic systemic autoimmune disease that predominantly affects women of childbearing age. The etiology of SLE is multifactorial and includes environmental, genetic and hormonal factors. SLE evolves by flares that can be triggered by environmental stimuli such as sun exposure or viral infections [1]. The disease can affect multiple organs such as skin, joints, central nervous system or kidneys. The pathophysiology of SLE involves a deficit in the clearance of apoptotic cells (debris) and the loss of tolerance towards nuclear antigens such as double stranded DNA (dsDNA), Smith antigen (Sm), ribonuclear proteins (RNP), Sjögren’s-syndrome-related antigen A and B (SSA/Ro and SSB/La) among others [2]. Activation of autoreactive T and B cells by these autoantigens leads to the development of autoantibody-secreting plasmablasts [3]. The resulting pathogenic autoantibodies are predominantly from IgG isotype, which will form circulating immune complexes (CIC) once associated with autoantigens and complement factors. These CICs will deposit in target organs and initiate a tissue-damaging inflammation amplified by the activation of various sets of innate and adaptive immune cells. Innate cells expressing activating receptors for the Fc portion of immunoglobulins (Fc receptors) and intracellular nucleic acid receptors, such as Toll-Like Receptor (TLR) 9 (dsDNA), TLR7 (RNA) and cyclic GMP-AMP synthase (cGAS)-stimulator of interferon genes (STING), are activated by CIC and produce pro-inflammatory cytokines amplifying autoantibody and CIC productions leading to the amplification of tissue damages [1,4].

These autoantibody and CIC-dependent pathways lead to the activation of several innate immune cell types. For instance, they trigger neutrophil and platelet activation to extrude DNA by NETosis (Neutrophil Extracellular Traps) and microparticles, respectively, increasing autoantigen bioavailability and CIC formation. Autoantibodies and CIC trigger the production by the monocyte/macrophage family of B cell activating factor of the TNF family (BAFF) that stimulates B cell maturation and thus, autoantibody production. They induce type I interferon (IFN) production by dendritic cells (especially plasmacytoid dendritic cells, (pDC)) also leading to an amplification of their own productions, and they allow basophil recruitment to secondary lymphoid organs where these cells amplify plasmablast number and function [5,6,7,8].

This simplified pathophysiology scheme of SLE underlines the key role of autoantibodies and CIC in flares and tissue damages occurring during disease course. The isotypes of the CIC-embedded autoantibodies will determine the cellular targets based on the expression by the latter of Fc receptors and the functional outcomes of such pathogenic compound binding. As reviewed before [2], autoreactive antibodies of IgG isotype are the most studied autoantibodies in SLE and have clearly been associated with disease activity and active lupus nephritis [2]. On the contrary, IgM isotype of autoreactive antibodies seems to have protective features against the development of tissue damage in SLE patients [2]. Autoantibodies of IgD and IgA isotypes have been described as well, but their pathogenic role in SLE still needs to be further characterized [2,9,10].

The present review will focus on the last discovered isotype of immunoglobulin, the least represented, namely IgE. As it will be discussed, the ability of IgE to regulate (directly or indirectly) the function of a number of immune cells and its strong activating features on IgE receptor-bearing cells give an immunoregulatory role to this isotype in the SLE environment that identifies it as a very promising therapeutic target.

## 2. IgE Receptors and IgE in SLE

Autoreactive IgE in SLE patient serum have been described over four decades ago [11] as was their ability to trigger basophil activation [12,13]. IgE is the least abundant isotype in serum immunoglobulins of healthy individuals (about 100 ng/mL of IgE to compare to circulating IgG concentration ranging from 5 to 10 mg/mL) and was discovered in the mid-sixties by Ishizaka et al. [14] as the main factor triggering immediate type I hypersensitivity. Elevated IgE titers reflects a type 2 immune response, i.e., a T helper (TH) type 2 (TH2) environment, as its production depends on type 2 cytokines IL-4, IL-5 and IL-13 [15]. IgE cannot activate complement pathways and their pathogenic role in allergic disorders is related to their binding ability to the [αβγ_2_] tetrameric form of the high affinity receptor for IgE (FcεRI) highly expressed by mast cells and basophils [16]. The FcεRI α chain binds IgE with high affinity (dissociation constant (Kd) = 10^−9^ mol/L), the β chain amplifies the downstream signal transduced by the dimer of γ chains through the phosphorylation of their ITAM (Immunotyrosine based activation motif) motives [16,17]. The FcεRI is found expressed at lower levels either constitutively or in an inducible way in a trimeric form [αγ_2_] on the surface of plasmacytoid dendritic cells (pDC), type 2 conventional dendritic cells (cDC2), Langerhans cells, monocytes/macrophages and eosinophils [16,18].

IgE can bind to its lower affinity receptor FcεRII/CD23 as well, a C-type lectin, known to regulate IgE synthesis either positively (soluble form, increased in SLE) or negatively (membrane-bound on B cells) [17,19]. FcεRII also exists in two isoforms: CD23a, expressed predominantly by B cells, and CD23b expressed also by B cells and by other cells such as T cells, dendritic cells, monocytes, Langerhans cells, eosinophils, platelets and gastrointestinal and respiratory epithelial cells [17].

The primary physiological role attributed to IgE is to confer protection against some parasite infections (either endo- or ecto-parasites) [20,21] and some environmentally harmful xenobiotics such as toxins or venoms [22]. As mentioned above, IgE was discovered as the main factor triggering immediate type I hypersensitivity [14]. Cross-linking of FcεRI-bound IgE to multivalent antigen (allergen), at the surface of basophils and/or mast cells, induces their activation and the release of pro-inflammatory molecules including vasoactive compounds (histamine), lipid mediators (prostaglandins, leukotrienes) and Th2 cytokines (like IL-4 and IL-13) that promote the development of a local or systemic allergic reaction (anaphylaxis) that can be lethal. Allergen-specific IgE will trigger FcεRI-bearing cell activation in allergic patients. On the contrary, allergen-specific IgG or more precisely an increased allergen-specific IgG/IgE ratio will reduce the allergic response in patients and may lead to allergen desensitization [23]. Immune complexes formed by the allergen coated predominantly with IgG and some IgE in lower amounts may co-engage FcγRIIB (inhibitory low affinity IgG receptor containing an ITIM (Immunotyrosine based inhibition motif) in its intracellular portion) with FcεRI, thus preventing, inhibiting or limiting FcεRI-bearing cell activation [24].

In atopic individuals, total IgE levels tend to be increased [25]. This feature has also been found in some cohorts of SLE patients; several groups studied the relationship between atopy and SLE with some controversial results [26,27,28,29,30]. In general, SLE patient cohorts show increased total IgE levels but accumulating evidence does not show any association between atopy and SLE [27]. However, in juvenile SLE, patients with atopic disorders show an increased disease activity and flare number as compared to non-atopic patients [31]. These data strongly suggest that total IgE and T helper (TH) type 2 (TH2) environment is associated with SLE and that IgE may play a role in disease pathogenesis. Some of our results from mouse models further underline this point. Indeed, three different spontaneous lupus-like mouse models (*Lyn^−/−^* [6], *FcγRIIb^−/−^* and *FcγRIIb^−/−^ × Yaa* [32]) were bred with IgE-deficient animals (*Igh7^−/−^*). In all three cases, IgE deficiency led to prevent, dampen or delay, respectively, the development of lupus-like nephritis and its associated immune activity [6,32]. These studies clearly established the immunoregulatory role of IgE in lupus pathogenesis. IgE titers in disease development may then be seen in different ways. First, as mentioned, they may reflect a systemic TH2 environment translating the pro-humoral context in SLE. Indeed, polyclonal hypergammaglobulinemia is a frequent condition in active SLE patients [33] and autoreactive antibody titers, particularly anti-dsDNA IgG, is associated with disease exacerbations [34]. Moreover, the analysis of *IL-4^−/−^ × Lyn^−/−^* mice, where the reduction in the global TH2 environment leads to a decrease in total IgE titers [35,36], also showed a dramatic decrease in lupus-like nephritis development [6]. Second, as for IgG, IgE should be considered both at the polyclonal level (total IgE levels) and also at the autoreactive level which translates a pathogenic function in the course of the disease. The following section will focus on autoreactive IgE prevalence and pathophysiological outcomes induced by such pathogenic factors.

## 3. Autoantibodies of IgE Isotype in SLE: Specificities and Prevalence

Evidence of antinuclear antibody of IgE isotype in SLE patients was discovered over four decades ago [11]. More precise specificities of such autoreactive IgE (to double stranded DNA (dsDNA), single stranded DNA (ssDNA), nuclear ribonucleoproteins (nRNPs) and Smith antigen (Sm)) were demonstrated through their ability to activate basophil degranulation ex vivo (Table 1) [12,13]. It is important to underline that autoreactive IgE specificity studies were based on known SLE-related autoreactive IgG specificities and realized on small patient cohorts [11,12,13,37]. As for IgG autoantibodies, dsDNA-specific IgE antibodies are the most prevalent and functionally studied autoantibody of IgE isotype in SLE (Table 1) [38]. We could demonstrate the presence of anti-dsDNA IgE in the *Lyn^−/−^*, *FcγRIIb^−/−^* and *FcγRIIb^−/−^ × Yaa* lupus-like mouse models as well as in a cohort of 42 SLE patients where their levels were associated with disease activity and especially with active lupus nephritis (Table 1) [6,32]. In 2014, we conducted a study on two independent cohorts of SLE patients to determine what the prevalence of autoreactive IgE was during the disease in order to appreciate its relevance to SLE pathogenesis and their pathogenic function (Table 1) [38]. We could demonstrate the high prevalence of autoreactive IgE found in 65% of the 196 SLE patients analyzed, and in 82% of SLE patients with active disease [38]. This prevalence was taking into account the four classical specificities of autoantibodies in SLE (dsDNA, SSA/Ro, SSB/La and Sm) as well as three new autoantigens identified in this study (CLIP4, APEX and MPG) (Table 1) [38]. Other recent studies by Henault et al. and Pan et al. also evidenced the presence of anti-dsDNA IgE in SLE patients and their functional outcomes on plasmacytoid dendritic cells and basophils, respectively (Table 1) [39,40]. The former study and a study by Khoryati et al. found, as we did, an association between dsDNA-IgE titers and SLE disease activity, and a high prevalence of dsDNA-IgE presence in their SLE patient cohort (Table 1) [6,38,39,41].

## 4. Autoreactive IgE and FcεRI-Bearing Cells in SLE

Several cell types express the FcεRI in humans (see above).

### 4.1. Mast Cells

Studies of mast cell contribution to SLE pathogenesis in human patients are limited and, to our knowledge, none have addressed their autoreactive IgE-mediated activation during the course of the disease. Mast cells accumulate in the tubulo-interstitial area of the renal cortex in SLE patients with lupus nephritis (LN) [42,43]. Recently, these infiltrating mast cells in LN patient biopsies were shown to be armed with IgE [44]. Whether their presence in this area contributes to lesion development, fibrosis development or repair mechanisms is not known. However, lupus-like mouse model studies demonstrated that mast cells do not play a role in lupus-like disease development and, if any, may be protective concerning skin lesions [6,45,46,47].

### 4.2. Basophils

**Figure 1:**
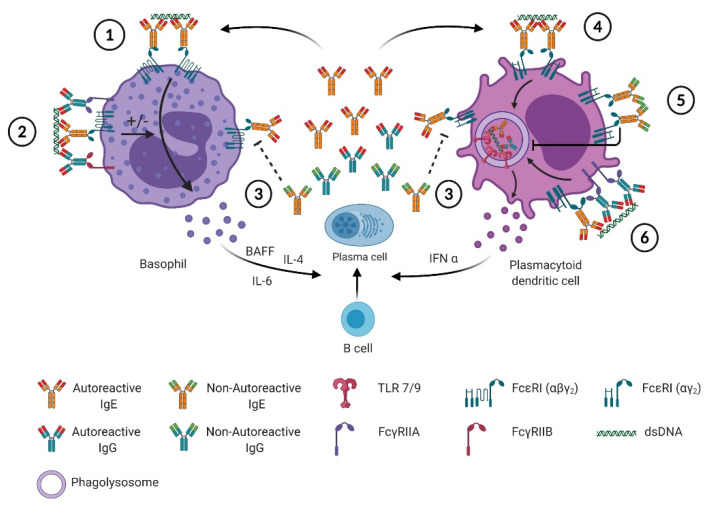
Autoreactive and non-autoreactive IgE effects on basophils and pDC in SLE. 1: The binding of the autoreactive IgE to dsDNA induces the crosslinking of the high affinity receptor for IgE (FcεRI) at the surface of basophils, which can lead to their degranulation and the secretion of lipid mediators and cytokines, such as: IL-4, IL-6 and BAFF. The secreted cytokines promote plasma cells differentiation and amplifies the secretion of autoreactive and non-autoreactive IgE/IgG. 2: In immune complexes, IgE and IgG autoreactive antibodies, together with the autoantigens, can aggregate both activating Fc receptors (FcεRI and FcγRIIA, respectively) and inhibiting Fc receptors, (FcγRIIB on basophils). This can modulate (enhance or inhibit) the activation signal mediated by autoreactive IgE through FcεRI and results in suboptimal activation where basophils do not degranulate but migrate to secondary lymphoid organs where they secrete cytokines influencing autoreactive B and T cell functions. 3: Non-autoreactive IgE can compete with autoreactive IgE for the binding of FcεRI, hence preventing the activation of basophils or pDC. 4,6: The binding of autoreactive IgE and IgG, respectively, to FcεRI and FcγRIIA on the surface of pDC, is followed by the delivery of these immune complexes to the phagolysosome where TLR9 is engaged and leads to the secretion of IFNα which promote B cell differentiation. 5: The crosslinking of FcεRI by non-autoreactive IgE inhibits the pDC activation by decreasing the FcεRI surface expression such as TLR7 and TLR9 expression and trafficking to phagolysosomes.

Basophils were the first cells shown to react to SLE autoantigens through an IgE-dependent mechanism in blood from SLE patients (Table 1) [12]. IgG only-containing immune complexes are not able to induce IL-4 production by murine basophils, unlike IgE only-containing immune complexes [6]. CIC purified from SLE patients can activate normal basophils and stimulate their IL-4 production, a feature lost once IgE antibodies are depleted from them (Table 1) [12,40]. Unlike others [40], we could not detect significant variation in CD63 expression on the surface of basophils from SLE patients, whatever the disease activity was, suggesting that autoreactive IgE antibodies, even during a flare of the disease, were not inducing basophil degranulation [7]. Constitutive basophil degranulation in SLE patients should induce some allergic-like symptoms chronically in patients, a feature that is not observed clinically. However, CD203c and CXCR4 expressions on SLE basophils and the extent of basopenia, correlating with disease activity in SLE patients, were witnessing a basophil “suboptimal” activation leading to their accumulation in secondary lymphoid organs [7]. One working hypothesis that may explain why basophils are not degranulating during flares of the disease is that CIC contain several isotypes of autoreactive antibodies including IgE and IgG (Figure 1). Then, CIC are able to engage and co-engage several Fc receptors either activating (FcεRI and FcγRIIA) or inhibiting (FcγRIIB) downstream signaling pathways. Integration of these positive and negative signals will determine the cellular response after such CIC engagement on the cell surface. Basophils are the leukocytes expressing the highest levels of FcγRIIB (ITIM-bearing (inhibitory) low affinity receptor for the Fc portion of IgG) and FcεRI/FcγRIIB co-engagement on human basophils is known to limit or prevent degranulation (Figure 1). [24,48]. Thus, increased basophil activation and extravasation during a flare of lupus may occur without degranulation whenever the balance between positive and negative signals is in favor of activation. Such switch may be induced by several factors. One factor could be increased autoreactive IgE concentrations in CICs, leading to an increase in the autoreactive IgE/IgG ratio, favoring a suboptimal activation of basophils. Other factors may be related to an increased expression of activating receptors (like FcεRI or FcγRIIA), a decreased expression of inhibitory receptors (like FcγRIIB) or another activating trigger (PGD_2_ or CXCL12 for instance). As previously mentioned, IgE is mandatory to get an optimal lupus-like disease development in various murine models and both autoreactive IgE and basophil activation are associated with disease activity in all tested mouse models and in SLE patients [6,7,32,38,40,49]. IgE-containing CIC and autoreactive IgE induce IL-4 production by basophils [6,40]. Since IL-4 positively regulates IgE class switch and total IgE levels in mice [36,50], IgE-mediated basophil activation during SLE may trigger a positive feedback loop leading to the amplification of the TH2 environment, the SLE disease activity and subsequent organ damage. Correlations between anti-dsDNA IgE titers, basophil activation (CD203c), basopenia and disease activity strongly suggest that this amplification loop contributes to SLE flares. Moreover, basophils accumulate in secondary lymphoid organs in SLE patients [6] and during the development of lupus-like disease in various mouse models where they amplify autoantibody production [6,7,40]. At least in the *Lyn^−/−^* mouse model, IgE deficiency prevents this accumulation, further suggesting the direct link between autoreactive IgE and basophil activation and accumulation in lymph nodes in the SLE context (Figure 1) [6].

### 4.3. Plasmacytoid Dendritic Cells

Type I interferons play a central role in the amplification of SLE disease activity and represent an active field of investigations in new therapeutic strategies for SLE patient care [51]. The cells described to produce large amounts of such mediators are the plasmacytoid dendritic cells (pDC) [52]. In 2016, Henault et al. described how self-reactive IgE (anti-dsDNA IgE) could activate IFNα production by pDC in a TLR9-dependent manner (Figure 1) [39]. Indeed, anti-DNA IgE and dsDNA-containing immune complexes lead to the delivery of dsDNA to TLR9 in the phagosomal compartment in an FcεRI-dependent way, inducing efficient IFNα production as anti-dsDNA-IgG + dsDNA complexes (Figure 1). This TLR9-dependent action of anti-dsDNA IgE is further enhanced when anti-dsDNA IgG antibodies are also present in the stimulating immune complexes (Figure 1) [39].

Previously, FcεRI crosslinking on pDC was shown to downregulate TLR9-induced IFNα production through a TNF-dependent autocrine pathway [53,54,55]. The same effect was observed on TLR7-mediated pDC activation, although TNF contribution to this phenomenon was not investigated [53]. In the latter study, the outcomes of such counterregulation was found to induce asthmatic patient (with increased total IgE levels) pDC to produce less IFNα in response to viral (influenza) stimulus [53]. In the SLE context, Khoryati et al. studied the effect of non-autoreactive (monoclonal) IgE on the ability of pDC from healthy individuals to produce IFNα in response to TLR7 or TLR9 stimuli and to ICs produced from SLE patient sera [41]. They showed that such IgE pre-incubation of pDC led the latter to reduce their TLR7- and TLR9-dependent IFNα production induced by SLE-derived ICs (Figure 1). The analyzed cohort of SLE patients showed a significant trend toward decreased IgE levels in active patients as compared to quiescent patients, unlike what was found in other studies [6,26,27,31,56]. As mentioned above, atopic juvenile SLE patients tend to have a more active disease than non-atopic patients [31]. Although this point has not been addressed yet by any study (to our knowledge), this feature may be due to increased anti-dsDNA IgE in these patients. Taking all these studies together suggests that a pro-humoral TH2 environment leads to increased total IgE levels in a given individual (Figure 1). As soon as FcεRI crosslinking on pDC occurs, without engaging TLRs, it dampens the ability of pDC to produce TLR7- and TLR9-induced IFNα via an autocrine TNF-dependent mechanism (Figure 1). This may contribute to the increased susceptibility of atopic patients to viral infections and the co-occurrence of viral infections with atopic disease exacerbations [57]. In the SLE context, increased autoreactive IgE content in CIC may counteract this inhibitory effect of higher total IgE levels by favoring dsDNA addressing to the phagosomal compartment of pDC to overstimulate TLR9-induced IFNα production (Figure 1). This working hypothesis may be tested ex vivo with the use of the tools developed by Henault et al. [39]. If validated, this hypothesis may also, at least partially, explain why anti-TNF therapies can induce SLE-like conditions [58]. Indeed, reducing the TNF-induced inhibition of TLR9-dependent IFNα production may lead to its exacerbation in a pro-autoimmune context. Evaluating total IgE levels in such patients before undergoing anti-TNF therapies may therefore be beneficial.

### 4.4. Other under-Investigated Actors

Anti-IgE and anti-FcεRIα autoantibodies have been described in the SLE context [6,59,60]. Although their contribution to the activation of FcεRI-bearing cells during SLE development has not been investigated, they may also contribute to the IgE-dependent amplification loop of the disease.

To our knowledge, another FcεRI-bearing cell type has not been studied in the SLE context concerning its ability to be activated by autoreactive IgE and by IgE-containing CIC: the type 2 conventional dendritic cells [18]. This potent antigen cross-presenting cell can produce high amounts of IL-12, IL-23, IL-1, TNF, IL-8 and IL-10 and respond to TLR7, TLR8 and TLR9 stimulations as well as other TLRs [61]. The analysis of their contribution to the autoreactive IgE-mediated amplification of lupus disease would be of great interest.

## 5. IgE-Oriented Therapies

All the data discussed above clearly identify autoreactive IgE and FcεRI-bearing cells as promising therapeutic targets in SLE. The present section will focus on some approaches targeting this axis, most of them being also developed in other autoimmune or atopic conditions where IgE and FcεRI-bearing cells are known to be essential in the pathogenesis, like chronic spontaneous urticaria (CSU), bullous pemphigoid, allergic asthma or atopic dermatitis [19,62,63,64,65].

### 5.1. Omalizumab

Preventing IgE binding to FcεRI would disable anti-dsDNA IgE-mediated activation of basophils and pDC as described above. Omalizumab is a humanized anti-human IgE monoclonal antibody that holds this feature. Approved by several drug administrations for use in asthma and chronic spontaneous urticaria, Omalizumab efficiently neutralizes circulating IgE, and then prevents their binding to FcεRI and lowers their circulating concentrations decreasing FcεRI expression on basophils, mast cells and dendritic cells [66,67,68,69]. This therapeutic opportunity may then reduce IgE-mediated amplification of autoantibody production driven by basophils and pDC. In a randomized phase Ib clinical trial, realized at the National Institute for Arthritis, Musculoskeletal and Skin diseases (NIAMS, NIH, Bethesda, MD, USA) by Hasni et al. [70], assessing the safety, tolerability and efficacy of Omalizumab in mild and moderate SLE patients, we showed that Omalizumab is well tolerated by SLE patients and is associated with an improvement of the disease, in terms of a reduction in the activity SLEDAI-2K score [71], and no worsening of the British Isles Lupus Assessment Group index (BILAG 2004) score [72]. A trend in the reduction in the type I IFN signature was observed in patients, especially those with a high baseline type I IFN signature [70]. These first very encouraging results, beyond the safety of Omalizumab in SLE, underline the need for larger clinical trials evaluating the efficacy of Omalizumab in SLE and validate the anti-IgE approach as a promising new therapeutic modality in SLE (Figure 1).

### 5.2. Ligelizumab

Ligelizumab is another anti-IgE humanized IgG1 monoclonal antibody developed more recently. Like Omalizumab, Ligelizumab binds to free IgE but with a higher affinity than Omalizumab (88-fold stronger affinity) and decreases more effectively IgE levels than Omalizumab [73,74]. Ligelizumab has been used in a randomized phase IIb clinical trial to test its efficacy for the treatment of chronic spontaneous urticaria (CSU) [63]. It showed a higher efficacy than Omalizumab to control patient symptoms [63]. Thus, the use of Ligelizumab for SLE patients may represent a better option than Omalizumab and may also break the IgE-dependent amplification loop of the disease (Figure 1).

Targeting directly IgE synthesis to avoid long term repetitive injections of biotherapies (that may lead ultimately to resistance) could represent an interesting option in SLE (see above) (Figure 1).

### 5.3. Quilizumab

New strategies limiting IgE synthesis have also recently emerged targeting the membrane-bound form of IgE on B cells. Indeed, another monoclonal IgG1 antibody has been developed, Quilizumab, that targets the M1 prime (M1′) transmembrane domain of IgE which is not present in secreted IgE [75]. The binding of Quilizumab on M1′ domain induces the apoptosis of the IgE-switched and IgE-bearing memory B cells lowering the levels of secreted IgE. Quilizumab have been tested on allergic rhinitis and mild allergic asthma and showed a reduction in serum IgE with an improvement of clinical responses to allergens [75]. However, in a randomized trial in adults with inadequately controlled allergic asthma, Quilizumab did not show clinically meaningful benefit on asthma exacerbations, lung functions, or patient-reported symptoms leading to the discontinuation of its development [76]. However, in allergic and autoimmune contexts, it may be beneficial to block the pathogenic IgE synthesis in order to prolong the effects of anti-IgE approaches to optimize therapeutic protocols for patients (Figure 1).

### 5.4. CSL362

Another approach to disrupt the IgE-mediated amplification loop of SLE would be to directly target the effector cells for which activation depends on IgE, namely basophils and pDC. CSL362 is a humanized anti-CD123 monoclonal antibody that prevents the binding of IL-3 to its receptor, thus inhibiting IL-3-dependent signaling, and that induces antibody-dependent cell-mediated cytotoxicity (ADCC) of CD123^hi^ cells [77]. Despite its original development for acute myeloid leukemia, Oon et al., in 2016, showed that CSL362 could deplete pDC and basophils ex vivo and then inhibit TLR9-induced IFNα production in peripheral blood mononuclear cells (PBMCs) from a cohort of SLE patients [77]. Subcutaneous administration of CSL362 in cynomolgus macaques depleted plasmacytoid dendritic cells and basophils and inhibited TLR9-induced IFN-inducible gene expression, strongly suggesting that CSL362 antibody and more widely basophil and pDC depletion may represent an interesting therapeutic option for SLE patients that requires further investigation (Figure 1) [77]. Of note, a phase Ib clinical trial aiming at evaluating CSL362 (JNJ-56022473, Talacotuzumab) safety and tolerability in SLE patients (NCT02847598) has been withdrawn before it started based on a change in the benefit risk assessment of this drug for the SLE population. Other antibodies or approaches targeting pDC and basophils or their released factors specifically are in development and may show significant benefits for SLE patients [78,79].

## 6. Concluding Remarks

Since their discovery, autoreactive IgE in SLE showed their pathogenic effects that are explained by their ability to activate both basophils and pDC. Their prevalence in SLE patients, their ability to overstimulate already dysregulated pathways in SLE and their association with disease activity clearly identify them as promising therapeutic targets. Development of clinical trials targeting this pathogenic entity and/or FcεRI-bearing cells should lead to real advancement in management of disease flares and SLE patient care.

## Figures and Tables

**Table 1:** Specificities of autoreactive IgE and their pathogenic role in systemic lupus erythematosus (SLE).

Autoreactive IgE Specificity	Prevalence (%)	Ref.	Activation of Basophils or pDC				Associated with	Ref.
			Basophils	Ref.	pDC	Ref.		
dsDNA	48.3	[37]	yes	[12]	yes	[39]	active LN and active disease	[6,38,39,40]
	35.4	[38]						
	54.4	[39]						
ssDNA	-	-	yes	[12]	NT	-	-	-
Sm	48.3	[37]	yes	[12]	NT	-	-	-
	7.5	[38]						
SSA/Ro	48.3	[37]	yes	[12]	NT	-		
	8.5	[38]						
SSB/La	6.9	[37]		[12]			mild and active LN	[38]
	4.1	[38]						
nRNP	62.1	[37]	yes	[12]	NT	-	-	-
Nucleosome	79.3	[37]						
CLIP4	10.0	[38]	NT	-	NT	-	-	-
APEX nuclease 1	0.2	[38]	NT	-	NT	-	-	-
MPG	0.2	[38]	NT	-	NT	-	-	-

Abbreviations used: dsDNA: double stranded DNA, ssDNA: single stranded DNA, Sm: Smith antigen, SSA/Ro: Sjögren Syndrome antigen A/Ro antigen, SSB/La: Sjögren Syndrome antigen B/La antigen, nRNP: nuclear ribonucleoprotein, CLIP4: CAP-GLY domain containing linker protein family member 4, APEX: AP endonuclease (apurinic/apyrimidinic), MPG: N-methylpurine-DNA glycosylase, LN: lupus nephritis, NT: not tested, pDC: plasmacytoid dendritic cell.
